# Properties, promotive and obstructive conditions of multi-professional teaching and learning of health professions and non-health professions: an explorative survey from the perspective of teachers

**DOI:** 10.3205/zma001025

**Published:** 2016-04-29

**Authors:** Daniela Schmitz, Ulrike Höhmann

**Affiliations:** 1 Witten/Herdecke University, Department of Nursing Science, Witten, Germany

**Keywords:** didactic concept, multi-professional learning, didactic methods, dementia, Master's programme

## Abstract

**Objective: **Care for people with dementia is considered a multi-professional challenge that requires a collaborative approach between health professionals and non-health professionals. Didactic strategies to ensure the same qualifications across these occupational groups are lacking. This article presents the joint learning of selected properties and promotive and obstructive conditions, using the example of a multi-professional Master's programme. It subsequently draws conclusions for didactic concepts.

**Methodology: **The perceptions of 12 teachers on this Master's programme, all representing different professions, were determined by using a qualitative exploratory survey on the three stated dimensions. With the aid of a summarising content analysis, their statements were condensed and abstracted so as to deduce appropriate requirements for methodical and didactic learning scenarios.

**Results: **In view of the fact that the students have very varied previous knowledge, the main challenge is finding a balance between expertise and tediousness. Establishing essential and common expertise, as well as sensitivity for different perspectives, is made particularly difficult by the fact that health and non-health professions differ greatly in terms of methods and approaches. For a successful outcome, the content focal points and didactic and methodical concepts for a learning group need to take into account the composition of that specific group. Recourse to didactic standard concepts is only possible to a limited extent.

**Conclusions: **The aim of joint teaching and learning of health and non-health professionals is to enhance the understanding of a profession: This is done by making individuals aware of their role in the chain of care, so they can recognise and organise the mutual conditionality of their own and external professional contributions.

## Zusammenfassung

**Zielsetzung: **Die Versorgung von Menschen mit Demenz gilt als multiprofessionelle Herausforderung, die eine kollaborative Gestaltung der Gesundheits- und Nicht-Gesundheitsberufe erfordert. Didaktische Konzepte zur gemeinsamen Qualifizierung dieser Berufsgruppen fehlen. Der Beitrag zeigt ausgewählte Besonderheiten, Barrieren und Bedingungsfaktoren deren gemeinsamen Lernens am Beispiel eines multiprofessionellen Masterstudiengangs auf und zieht Konsequenzen für didaktische Konzepte.

**Methodik: **Die Einschätzungen von 12 Lehrenden dieses Masterstudiengangs, die unterschiedliche Professionen repräsentieren, wurden mit einer qualitativ-explorativen Befragung zu den genannten drei Dimensionen ermittelt. Mit Hilfe einer zusammenfassenden Inhaltsanalyse wurden deren Aussagen kondensiert und abstrahiert, um Anforderungen an geeignete methodisch-didaktische Lernszenarien abzuleiten.

**Ergebnisse: **Vor dem Hintergrund sehr divergierenden Vorwissens der Studierenden besteht die zentrale Herausforderung darin, eine Balance zwischen Expertise und Langeweile zu finden. Das Herstellen notwendiger gemeinsamer Gegenstandsverständnisse und Sensibilität für unterschiedliche Perspektiven ist besonders dadurch erschwert, dass Gesundheits- und Nicht-Gesundheitsberufe sich in ihren Arbeits- und Herangehensweisen stark unterscheiden. Zur Zielerreichung sind je nach Zusammensetzung der Lerngruppe unterschiedlich konturierte inhaltliche Schwerpunkte und didaktisch-methodische Konzepte notwendig. Ein Rückgriff auf didaktische Regelkonzepte ist nur begrenzt möglich.

**Schlussfolgerungen: **Das gemeinsame Lehren und Lernen von Gesundheits- und Nicht-Gesundheitsberufen zielt auf ein erweitertes Berufsverständnis ab: Durch die Wahrnehmung als Teil der Versorgungskette, die wechselseitige Bedingtheit der eigenen und fremden Professionsbeiträge zu erkennen und zu gestalten.

## 1. Challenge: Care for people with dementia and didactic issues

Care for people with dementia (PWD) is an organisational task that can only succeed when various occupational groups work together. In doing so, individuals in health professions (HP) must coordinate their action plans, both strategically and in terms of content. They must also always integrate their contributions into the requirements, parameters and consequences of the actions of framework-setting non-health professions (NHP). This is because the direct care provided is always subject to conditions that are established by others, and these actions in turn have consequences for others. For this to be carried out systematically, both the HP and the NHP need to broaden their professional perspectives. At the same time, it should be noted that across different professions' bodies of knowledge and job requirements, the topic of "dementia" is placed on different hierarchical levels. For caregivers, for example, precise and appropriate care in their daily interactions with PWD is of utmost relevance. An architect's work is "only" part of a broader context. For an architect, this takes the form of specific interior design requirements and parameters of a building project. For one's concrete everyday working life, interaction with PWD is only of marginal importance. In order to strengthen collaboration, HP and NHP need to learn to recognise interrelations when dealing with patients and their relatives, as well as with organisations and societal parameters of care.

### The concept of the Master's programme

The multi-professional in-service Master's programme "Caring for People with Dementia" at the Witten/Herdecke University prepares students for these demands. The course's aim is to train "change agents", who develop a broader understanding of their professional practice and can then purposefully coordinate their work with other professions. In German, change agents are referred to as "Veränderungsagenten" in the Sociology of Professions [1].

Students represent both HP and NHP. They exercise professional activities that are flexible in terms of organisational possibilities. Students are primarily: Nursing scientists, therapists, educators, social workers, as well as medical professionals, economists, technicians, psychologists, historians, journalists, theologians, architects and building designers.

The skills acquired during the programme enable graduates to deal with frequent and unpredictabe changes in fields that organise and provide care. The students acquire professional expertise that help them identify the key care-related issues in their fields, and help them coordinate their efforts with other actors and their interests. On this basis they work on multi-professional problem-solving, while taking into account the interrelations when dealing with patients and their relatives, as well as with organisations and societal parameters of care. They are able to implement their solution approaches and evaluate them. All occupational groups learn how to make their professional contributions compatible with other professions [2]. The curriculum describes a bottom-up learning pathway from the micro to the macro level. First a common basic understanding of the phenomenon of dementia as well as nursing and therapeutic basic concepts are developed. Next institutional aspects and communal concepts of care are looked at, which finally lead into the demands that are linked to politics and society as whole. 

Professionals choose this programme for its flexible self-study phases [3] and the multi-professional learning aspect. They associate individualised skills development with the course. The following example illustrates how joint solutions can be found: An architect, an occupational therapist and a business economist must develop a concept for the lay-out of a common activities room in a new in-patient care facility. The first discussions are about issues related to the colours of the walls and ceiling, the floor covering, the lighting and windows, the furniture and whether the carers need a direct view into the room. Should there be a built-in kitchen or not? Are there any hygiene concerns? After a short space of time the group realises that each member has a different type of resident in mind. Everyone has a different view on how the people can best spend their day there. The following becomes clear: To decide on the design of the room, not only must the overall concept of the care facility be looked into, but the differentiated parameters of the work procedures and specific requirements must also be taken into consideration. 

The joint learning offered to HP and NHP is what makes this programme unique in Germany. This is because care for PWD requires an understanding of the organisational and societal parameters for HP, as well as of how these come about and are configured. Moreover, framework-setting NHP must know what it is like to live with dementia and what care concepts look like in order to understand the consequences of one's own decisions and to create precise and appropriate structures.

#### 1.1. Definition of the subject matter: Didactics for multi-professional teaching and learning contexts 

Multi-professional course units are becoming increasingly compulsory for medical, therapeutic and care-related study programmes. Didactic concepts have so far only focused on joint learning of health professions and the integration of their respective contributions in professional interactions [4], [5], [6], [7], [8], [9], [10], [11], [12]. This is not sufficient for caring for PWD. Contributions from NHP, whose requirements have consequences for the work of HP, need to be interconnected with the latter. This particularity also means that special didactic concepts are necessary to ensure that all students are equally supported and included. As yet there has been a lack of didactic concepts [13], as well as a lack of a clear understanding of the term "multi-professionalism" [14].

Constructivist didactics are used as a framework model in the programme. Teaching and learning should be understood as individual social constructions. Social constructions from various perspectives are therefore possible. Within these a shared reality which serves as a joint understanding of the subject matter needs to be agreed upon [15]. The challenges of didactic design lie in the bridging between HP and NHP perspectives [16] and in the inevitable encounters between these relatively highly specialised occupational groups. Both have different approaches, coordination requirements and specialised replacement needs with regard to the care for PWD [17]. The complexity results from the interdisciplinary aspect of the learning content and the heterogeneous professions of the teachers and students. The teachers need highly specialised and reflexive skills. They must possess a transdisciplinary approach so as to be able to think beyond their own subjects and the limits of their own professions [18]. They need to reflect on methods and modes of thought from other subject areas, integrate different perspectives, assay syntheses with teachers of other professions, and process these in order to impart learning content. The subjects should be imparted after they are transformed beyond the traditional limits of their discipline [19], [20].

If students can be didactically engaged using their current situation as a starting point, the benefit of joint learning lies in the fact that both groups can mutually integrate their professional contributions on various levels. Medical professionals do not generally acquire basic knowledge of architecture or urban planning during their studies, and conversely an architect does not receive training in therapeutic fundamentals. Joint knowledge makes it easier for those concerned to decide on appropriate care and parameters. 

The aim of this article is to highlight the properties, as well as the promotive and obstructive conditions of joint learning of health professions and non-health professions, and to subsequently deduce design factors for joint learning arrangements.

#### 1.2. Literature survey

By means of a systematic literature search, promotive and obstructive parameters of multi-professional learning could be determined. Since multi-professionalism goes beyond health professions in existing literature, research was undertaken using specialist databanks like FIS Bildung, Wiso Sozial- und Wirtschaftswissenschaften, PSYNDEX, EBSCO, google scholar and PubMed. Finally, other relevant articles were identified using a snowball system. Experience reports, written statements and practice-based project descriptions were excluded. Out of over 500 articles, 96 points of reference for didactically relevant advantageous and obstructive factors were found. Content reviews revealed that 30 of these included concrete analyses. In general, multi-professionalism in medical or nursing contexts takes two or three professions into account. In social work, sometimes healthcare professionals, therapists and caregivers also belong to the multiprofessional team with social workers and administrative workers. The framework that is the most comprehensive in our understanding of the concept is that of school development and child day care. Here framework-setting occupational groups in the fields of housekeeping, building services, politics, public services and administration, as well as parents and others concerned are considered part of a multi-professional team [21], [22]. 

Obstructive factors originate in professional practices, and are normally the result of poor knowledge, and of presuppositions about other professions that are culturally predetermined or reproduced on a daily basis [23], [24]. In teaching and learning contexts these can have an effect on personal, interpersonal or organisational levels. A failure to appreciate one's own professional role in practice, as well as a lack of occupational safety, can have a negative impact on a personal level [25]. A lack of team spirit, different bodies of knowledge, language (in engineering, for example, the phrase "state of technology" is used instead of "state of research") and modes of thought have an obstructive effect on an interpersonal level [26], [27]. Varying working styles and attitudes to work, unrealistic expectations of groupwork, and a lack of agreement in relation to teamwork can all be obstacles to joint learning [23]. Sometimes power and interpretative hierarchies arise between individual professions and their respective focal points [8], [10]. On an organisational level other tasks, understandings of situations and targeted activities [28], [29] complicate collaboration.

Promotive factors for multi-professional teaching and learning can have an impact on personal and organisational levels, and can also affect joint learning processes. On a personal level, the heterogeneity of individuals' practical knowledge, training and professional socialisation must be made known and also compatible. To this end, there needs to be a common language and open interaction. For the latter, time and space need to be made available. As for joint learning processes, the important issue is the imparting of knowledge regarding tasks, authority and responsibilities of the professions involved. Upon becoming better acquainted with one another, those involved are able to correct erroneous presuppositions. Promotive factors in inter-professional training lie in the joint and positive learning experiences. They also enable participants to share experiences, they eliminate differences in status, offer opportunities for reflexion, prepare participants for collaborative teamwork, and help cultivate all of the above [23], [16], [4], [6].

Once the factors are established, the question arises of which promotive and obstructive factors also apply to teaching and learning contexts of HP and NHP, and which are the additional specific factors. This is where the exploratory investigation begins. The results of the research are used for comparison and to classify the statements of respondents. The leading research hypothesis for the survey was that, on the one hand, a large number of the promotive and obstructive factors are also relevant to this context and that, on the other hand, due to the broader group of actors, some factors need to be qualified and new ones also apply.

## 2. Method – qualitative exploratory survey

### Research gap and procedure

The qualitative exploratory survey should contribute to reducing the research gap regarding joint learning of HP and NHP, which are expected to have an effect on the chain of care. Previous research approaches have only looked at the joint learning of two or three HP. For that reason, an exploratory, qualitative and written survey for teachers was developed for the joint learning of these occupational groups. It was carried out from 05.02-22.02.2015. The explorative approach takes the lacking, specific didactical concepts for this particular educational setting into account. 

#### Sample

Survey recipients were teachers who had been working for the programme on a long-term basis (n=23), and who had taught for the programme for at least three semesters since it began in 2012. The teachers were contacted by e-mail. 

##### Material

Using the existing literature as a base, the teachers were presented with 6 written questions that related to key didactic issues of multi-professional teaching and practice. The questions were each subdivided into 3 questions to allow for descriptions of the phenomenon of multi-professionalism, as well as assessments of promotive and obstructive factors in teaching and learning. For example: "In your interactions with students, in which contexts do you notice their different multi-professional backgrounds? Please give examples." and "In your experience while teaching, in which situations did multi-professionalism promote joint learning processes?"; factors that obstructed processes were also asked about. Asking about concrete situations makes it easier for respondents to recall concrete experiences. The teachers formulated their answers giving examples in sentence form using the Word template, and then sent them back via e-mail.

##### Analysis

The survey data was assessed by means of a Mayring content analysis using a deductive summarising technique [30], in which statements were paraphrased and condensed into categories. To synthesise the processed data, dimensionally summarised requirements for a didactic concept were then deduced. These requirements are considered significant for supporting all professions in the same way, integrating them into the learning process and maintaining everybody's interests in the overall process. The presentation of the results is based on indicators of properties, and of promotive and obstructive conditions.

## 3. Selected results

After a participation reminder, the response rate of the survey was 52%, which meant 12 teachers. 

The teachers asked in advance to provide information about their professions. Some assigned themselves more than one profession because they had additional qualifications. The number of profession assignments in Table 1 (Tab. 1) is therefore higher than the number of respondents. 5 of the 12 teachers assigned a total of 13 different professions to themselves, and thus possess an "inherent" multi-professionalism.

Next, specific factors of multi-professionalism from the perspectives of the teachers are presented. These go beyond the general influencing variables stated in the literature.

### 3.1. "Differentiated previous knowledge and professional tasks: the tediousness of highly specific specialist knowledge" – Properties of multi-professional teaching and learning (11 mentions from 7 teachers)

The perception and description of the phenomenon of multi-professionalism for most of the teachers is characterised by the essential need that all teachers feel to find a balance between tediousness and individually specific expertise. Content also needs to be relevant for all occupational fields. The teachers emphasise that different levels of knowledge lead to different expert roles, which the teachers then need to incorporate into their teaching. 

These varying *levels of knowledge and heterogeneous fields of relevance* become apparent, for example, in different content-relevant categories of dealings with the phenomenon of dementia. The results show that students use profession-specific categories when they discuss and present course content, and also use profession-specific assessment criteria: "if a student has a background in nursing science, she will initially use technical terms when describing an example, and reasons using concepts from her specialised background" (Teacher 6).

Therefore the students, depending on the learning content, are *sometimes experts and sometimes novices* in individual fields of knowledge. Each profession plays both roles in the programme. It depends on which field of knowledge is being focused on, and on how deep and broad the professional previous knowledge is. This is described by Teacher 3 using the following example: "there are big differences in knowledge about and openness towards economic thinking and technical solutions to support care work." A positive attitude on the part of the students towards individual course units is necessary here. This can be seen in their openness towards knowledge and notions that are foreign to their fields. They also must accept content that may only be marginally directly relevant to their occupational groups. At the same time, students have to accept that when it comes to content that is specific to their fields, they will "only" update their levels of knowledge. 

The multi-professionalism of students is seen in the results, and especially in the differences: They have *different conceptions and skills with regard to scientific work and the understanding of their professions.* Their working styles vary in terms of autonomy, research skills, processing of content as well as presentation techniques. For all profession-related differences, the reference value of the number of students in each cohort (around 15) is not sufficient to be able to indisputably attribute these differences to the disciplines that the students were initially trained in.

The survey showed that from a didactic perspective and from the teachers' point of view , individual professions can be capitalised by establishing specific profession-related anchors that serve as connecting factors. For example, the PICOS schema was mentioned by teachers as being a profession-related anchor for medicine and nursing, and sometimes for therapists, in research-related topics. Outside of medicine and nursing, that is, in non-health professions there is no need for this. This is due to profession-specific research logic and the complexity of research questions. Another example from the results is the subject of relationship structures. In this regard relationship dynamics and early childhood relationship experiences serve as an anchor for psychologists, educators and social workers. An anchor for architects and engineers is social design. Depending on the learning content, teachers and students can address profession-specific aspects as novices or experts, discuss them together in a problem-solving way with the occupational groups, and externalise profession-specific matters of fact. 

#### 3.2. "Develop common understanding" - promotive conditions of multi-professional teaching and learning (9 mentions from 8 teachers)

According to the teachers, for the fostering of multi-professional learning processes it is important to create opportunities for the different students to make specific contributions based on their *profession-related individual experiences, actions and expertise.* For example, "in discussions during the seminar, nursing concepts that are very familiar to most students are mentioned. Students from other disciplines ask about the concepts and wish to have them explained. In this context, the explanation helps everyone develop a common understanding of the concept. Often it becomes obvious that seemingly shared fundamental knowledge was not as clear as it was thought to be." (T6) *Further developing one's own professional expertise and aligning this at the same time with other perspectives is a main priority here.* Dealing with complex problems and joint project work were also rated as particularly suitable in the results. Joint learning is further promoted through a targeted and animated changing of roles, which involves students with expert knowledge also becoming teachers for certain content. Here the issue is about knowing concepts from other professions and making one's own view of problems compatible. It is also important to define requirements, consequences and action parameters. Another example from the results is that teachers seek all perspectives in regard to the learning content, and should encourage students to compare and expand their perspectives. The aim here should be to develop a *common understanding of the learning content. *

#### 3.3. "How much do I need to know about it, although I'm not an architect?" –Barriers to multi-professional teaching and learning (12 mentions from 11 teachers)

Teachers also state factors that can make multi-professional learning processes difficult. These concern the well-known problems that arise when individuals do not manage to establish mutual respect for and trust in others' abilities, and differences in the power of interpretation, trust, hierarchies and specialist fields of the respective professions cannot be bridged. At the same time, three teachers indicated that this turned out to be less difficult in classroom interactions than in everyday working situations. In everyday working situations, it is more common for people to step back than to look for compatibility. From this we can deduce that things often depend on the *everyday working lives of the students.* Study exercises that are didactically laid out in the curriculum *can only have a limited influence* on whether the economic relevance of the acquirement of knowledge becomes an interest benchmark, instead of the description of concrete care tasks. 

The composition of learning groups is particularly seen as a determining factor among the teachers in the survey: Four teachers point to the significance of approximate *quantitative equilibria of the professions* in the learning groups. "When the majority of the students come from one discipline and only a few others belong to other disciplines, it often becomes tedious to repeatedly explain concepts to individual students that the majority are already familiar with. When many disciplines are represented, there is a much greater willingness to become involved in new and different concepts, and to explain these. Then there is usually a consensus that people can learn from each other. In the first case, there is a greater impression that individuals have to 'go along with it.'" (Example from Teacher 6). It can be different when it comes to non-fundamental knowledge. Profession-specific content can be delegated to the respective profession-experts, in the sense that everyone holds some responsibility, but this is where one's own responsibility stops. In discussions or groupwork phases, reservations or know-all attitudes can block learning processes or even lead to a situation in which there is no agreement and no jointly coordinated result. Finally, learning processes can be slowed down if demarcations are constantly redefined and if roles need to be discussed frequently. 

The survey shows that teachers in situations that cannot be structurally modified have to look for acceptable rules and compensation mechanisms for the group. Dynamic teaching and learning methods can be helpful, that is, methods that strengthen the roles of individuals in the groupwork phases. In addition to the consideration of individuality, what also plays a role is how well teachers manage to take the cultural characters of their students' professions into account. In order to mitigate distinct obstructive factors, teachers must be able to show a high degree of personal initiative, and the learning content must arouse curiosity among all professions. There are no didactic standard concepts for these particular learning situations. 

## 4. Discussion

In this section we focus on the promotive factors of multi-professional learning of HP and NHP (a&b) and discuss the associated didactic implications for studies and methods for multi-professional learning sequences (c&d).

### a) Structure and adjustment of shared knowledge bases

The structure of a shared knowledge base is formed by *profession-specific linguistic* *categories.* Teachers need to work out that, for example, economists and architects call on other treatment relevant categories to the description of the phenomenon of dementia, such as caregivers and doctors. Implicit professional matters of fact, however, according to H. Garfinkel's ethnomethodology, only become known when others go against them when the point is being discussed. On that point, suitable controlled "contraventions" to profession-specific matters can be purposefully brought about and made productively useful for the group. 

In addition these differences need to be mutually comprehensible and common understandings of purpose need to be established. Thus for an architect, for example, the subject of orientation in space is of key importance. For nursing staff, it is rather daily dealings with defiant behaviour. The composition of a learning group is a determining factor. Despite curriculum specifications, it is difficult to determine in advance which occupational fields, experiences, knowledge bases and orientations the students will bring with them, and which of these merit particular attention as parameters. 

A common reality construction [15] to serve as a "common boundary of understanding" [31] must be agreed upon. To this end, fundamental texts which help prepare students for classes are advisable for all participants, because a medical professional generally learns nothing about building design in his/her studies. Working with fundamental texts can happen because the lack of basic knowledge of many professions is made up for by their practical knowledge. It is fundamentally different to learning in undergraduate programmes, and here it can be productively used as a central method for shared learning. The breadth and depth of joint topic discussion can be different depending on the composition of the learning group. In the first step it is necessary to establish a common body of knowledge that includes the varying perspectives and actively makes participants aware of unchallenged guiding concepts. In the care for PWD, these requirements are associated with HP and NHP who are concerned with how PWD live in the community, and how shops and neighbourhoods can be incorporated, with the aim of, for example, avoiding self-endangerment.

#### b) Reflective practical knowledge and changing roles

The survey of teachers made clear that with regard to the promotive factors, *role-changing* between students and teachers was significant. So was the *contribution of practical knowledge that came about from reflexion.* In terms of method, this can be initiated by questions that clear up confusions regarding the learning content and the understanding of terms. The professionals are constantly invited to describe their specialised expertise as if they were perceiving it from the outside, and to describe the effects that their own actions have on others. To make comparisons, the concerned professions are asked [21], [22] which concrete effects they experience and to what extent both perceptions coincide. Moreover, the individual social constructions [36] are identified through dialogue. They are identified as reciprocal requirements, parameters and consequences of respective actions.

##### c) Special relevance of project-based learning

Within the framework of multi-professional project-based learning, profession-related categories and commonalities in research processes become evident [23], [32]. Project-based learning is structured using a research cycle [33]: First a question that makes sense to all participants must be developed. Next, a common understanding of the problem and subject matter using a shared analytical orientation framework is developed. After comparing the varying research understandings, a research design, to make sure there is a common approach, is determined. In project work, the students can discuss problem-solving from various angles, and develop and test compatible implementation strategies. They thus acquire skills to reflexively explain profession-related working styles and procedural techniques (e.g. in project development), and to define goals within development projects. Continuous and guided reflection should therefore be a part of project-based learning in all its phases. The value of project-based learning lies in the mastering of reciprocal coordination processes and finding common solutions, which ideally represent the specialised expertise of the professions involved [34]. In addition, the relevance of requirements and consequences of actions of other professions in a practically real professional environment become clear. Real collaboration conditions are also taken into account - in contrast to other multi-professional learning that ignores everyday realities.

##### d) Methods for multi-professional learning sequences

If very common teaching and learning methods are analysed taking into account the demands for perspective adjustments, the following examples are suitable for multi-professional learning: collegial consulting, creativity techniques, simulation games, podium discussions or multi-perspective case studies. Project work, research-based learning, creativity techniques, podium discussions, simulation games and case studies should therefore be included in the toolboxes of teachers who work with multi-professional learning groups. An quintessential learning sequence is a multi-professional podium discussion. In the preparation for this, individual perspectives are enhanced, tasks and responsibilities are negotiated, and there is an exchange of expectations and objectives. In the discussion, conditions of one's own actions and consequences for the actions of others become apparent. Profession-specific viewpoints and matters of fact can be identified in the analysis and reflection phases of the discussion. The aim of multi-professional learning sequences must be to broaden students' perspectives and ultimately to promote communication between individual social constructions.

## 5. Conclusions

For needs-based care for PWD, the most important factor is ultimately the mastering of a systematic multi-professional interconnection of perspectives. This can be seen when looking at requirements and parameters that other occupational groups create for their own actions, as well as in the sensitivity and perception of consequences of one's own actions for others. The highest teaching objective of multi-professional learning is therefore the realisation that actions are always affected by others' conditions and also have an impact on others. This premise makes it possible to appreciate the advantages brought by heterogeneous occupational groups, and to impart complex knowledge regarding problem-oriented care. Only when the occupational groups integrate the understandings of their professions into societal realities and working conditions can they bring together relevant requirements into this understanding.

Due to the small sample size, there are limitations to the transferability of these didactic approaches to other teaching and learning contexts. Nevertheless, approaches of individual methods can still be employed in other teaching and learning contexts that involve two or three professions [23], [9], [11], [12], [35], [36], [37]. However, the concept components for multi-professional teaching and learning of HP and NHP that are presented here are still relevant. The aim is to come up with a comprehensive understanding of the care-related design requirements and problem-solving between the two groups [2]. 

Limitations arise due to the exploratory nature of the issue. Fundamentally, there are similar results in multi-professional contexts concerning occupational groups within one field. HOWEVER, here there are additional challenges, such as varying professional understandings of the phenomenon of care for PWD. In addition, all professionals need to learn that their everyday work can only function as part of an interdependent network of personal, organisational and greater societal requirements. They must constantly allow themselves to think beyond their own practical work and consider care contexts and chains of action, and thus develop a new, broader understanding of their profession.

## Competing interests

The authors declare that they have no competing interests.

## Figures and Tables

**Table 1:**
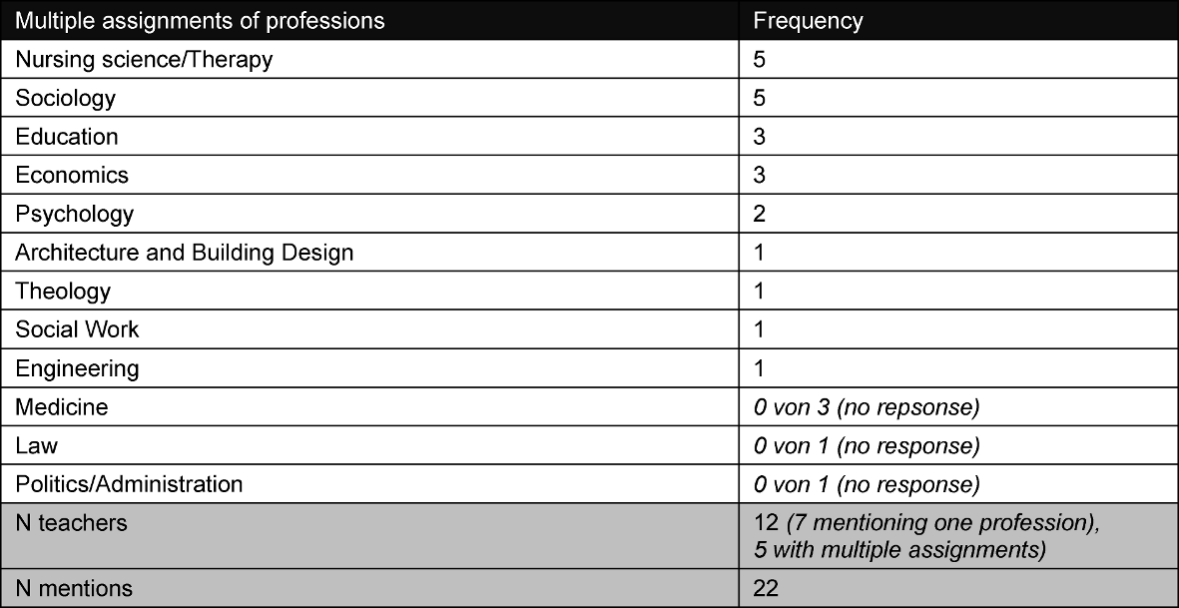
Professions of the 12 teachers (N=20, multiple assignments)
